# Adiponectin protects the rats liver against chronic intermittent hypoxia induced injury through AMP-activated protein kinase pathway

**DOI:** 10.1038/srep34151

**Published:** 2016-09-28

**Authors:** Wenxiao Ding, Qiang Zhang, Yanbin Dong, Ning Ding, Hanpeng Huang, Xianji Zhu, Sean Hutchinson, Xingya Gao, Xilong Zhang

**Affiliations:** 1 Department of Respirology, The First Affiliated Hospital with Nanjing Medical University, 300 Guangzhou Road, Nanjing 210029, China; 2 Morsani College of Medicine, 12901 Bruce B Downs Blvd, Tampa, FL 33612, U.S.A; 3 Department of Physiology, Nanjing Medical University, 140 Hanzhong Road, Nanjing 210029, China

## Abstract

This study was performed to assess the effect of chronic intermittent hypoxia (CIH) on the liver, the associated mechanisms and the potential therapeutic roles of adiponectin (Ad). Sixty rats were randomly assigned to four groups: the normal control (NC), NC and Ad supplement (NC + Ad), CIH, and CIH and Ad supplement (CIH + Ad) groups. The rats in the CIH and CIH + Ad groups were exposed to a hypoxic environment for 4 months. Rats in the NC + Ad and CIH + Ad groups were also treated with an intravenous injection of Ad (10 ug), twice a week. The plasma levels of hepatic enzymes, serum triglyceride, liver triglyceride, fasting blood glucose and hepatic cell apoptosis in hepatic tissue, were higher in the CIH group than in the NC and NC + Ad groups. However, the Ad supplementation in the CIH + Ad group rescued the hepatic tissue insult by activating the AMP-activated protein kinase (AMPK) pathway. In conclusion, Ad could protect against CIH-induced hepatic injury partly through the AMPK pathway.

Obstructive sleep apnea syndrome (OSAS) is a common disease that affects 2–4% of adults^1^. OSAS is associated with numerous pathologies, such as cardiovascular disease^2^,^3^, metabolic syndrome^4^,^5^, diabetes mellitus^6^, and dyslipidemia^7^. OSAS is associated with an increased risk of non-alcoholic fatty liver disease, non-alcoholic steatohepatitis and fibrosis^8^. Valerio Nobili *et al.* have reported that OSAS in children is associated with increased risk and severity of non-alcoholic steatohepatitis, independently of obesity and insulin resistance^9^. Additionally, chronic intermittent hypoxia (CIH) exacerbates endothelial dysfunction in cirrhotic rats^10^. CIH is one of the most important characteristics of OSAS. Savransky, V *et al.* found CIH could lead to mild liver injury in Lean C57BL/6J mice without obesity^11^. However, the effect of CIH alone on hepatic tissue is still unknown.

The endoplasmic reticulum (ER) is an important subcellular effector that maintains cellular homeostasis. When stimulators disrupt cell homeostasis, abundant unfolded or misfolded proteins accumulate in the organelle, leading to additional ER stress^12^,^13^,^14^. To manage the accumulation of these proteins, cells activate a specific adaptive response called the unfolded protein response (UPR). The UPR has three pathways: the inositol-requiring enzyme 1 (IRE1) pathway, the PKR-like ER kinase (PERK) pathway and the activating transcription factor (ATF6) pathway. When the stimulation is excessive, the UPR can result in cell death^15^. ER stress plays an important role in NAFLD in humans liver^16^ and the liver transplantation in rats^17^. Furthermore, our lab has shown that CIH can induce ER stress in myocardial tissue and in the genioglossus^18^,^19^. Therefore, we hypothesized that ER stress might be involved in the hepatic injury induced by CIH.

Adiponectin (Ad), an adipokine synthesized and excreted into the blood by adipose tissue, plays an important role in energy metabolism and adipocyte differentiation^20^,^21^,^22^. Multiple studies have shown that adiponectin plays a protective role *in vivo*^23^,^24^,^25^. Yamauchi *et al.* reported that Ad could inhibit liver gluconeogenesis^21^. Our previous study also revealed that Ad could alleviate the genioglossal mitochondrial injury in rats exposed to CIH^26^. However, the role of Ad in hepatic tissue under CIH conditions is still unknown. The aim of this study was to assess the effect of CIH alone on the liver and the protective roles of Ad as well as to uncover the underlying mechanisms.

## Results

### Biochemical changes

To study the effect of CIH on the liver, we assessed the liver triglyceride, fasting blood glucose (FBG) and the serum levels of alanine amino transferase (ALT), aspartate amino transferase (AST), lactate dehydrogenase (LDH), alkaline phosphatase (ALP), triglyceride and fasting insulin (FSI). The liver triglyceride and the serum levels of ALT, AST, LDH, ALP and triglyceride were higher in the CIH group than in the normal control (NC) and NC and Ad supplement (NC + Ad) groups, whereas those in the CIH and Ad supplement (CIH + Ad) group were significantly lower than those in the CIH group but also higher than those in the NC and NC + Ad groups (Table 1). These findings were all statistically significant (p < 0.05). No significant differences were found between the NC and NC + Ad groups (p > 0.05). Among the four groups the FBG level in the CIH group was significantly higher than in other three groups (all p < 0.05), while FBG levels in NC, NC + Ad and CIH + Ad groups showed no statistical difference (all p > 0.05) (Table 1). The level of FSI was the lowest in the CIH group among four groups (Table 1). The FSI level in the CIH + Ad group was higher than in the CIH group but lower than in the NC and NC + Ad group (all p < 0.05). There was no significant difference between the NC and NC + Ad groups (p > 0.05).

### Histological changes

**Figure 1:**
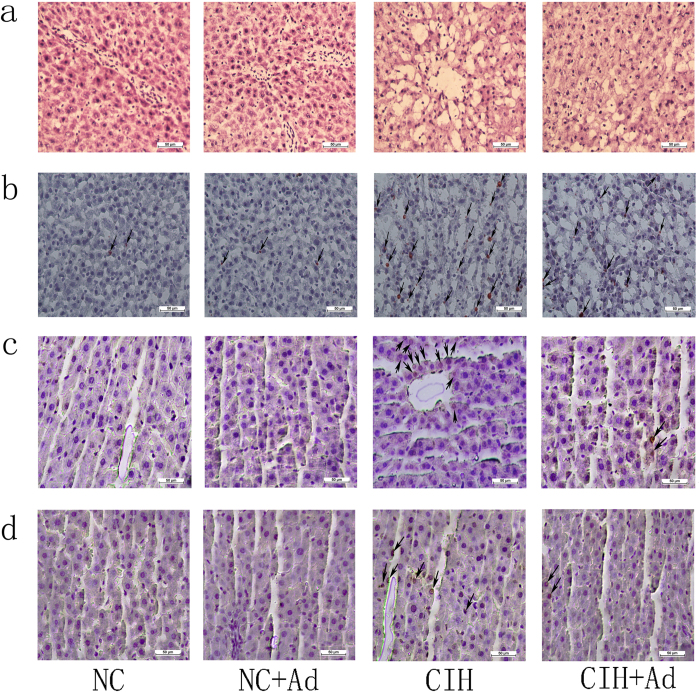
Histological changes. (**a**) The morphology of hepatic cells in four groups detected by HE staining (original magnification X 400). (**b**) The lipid staining of hepatic in four groups detected by Oil red O staining (original magnification X 400). (**c**) The hepatic cell apoptosis detected by the TUNEL staining (original magnification X 400). (**d**) The ki-67 staining for hepatic proliferation in four groups (original magnification X 400). The arrow points the positive cell.

The hematoxylin-eosin (HE) staining showed that the hepatic morphology changed with large vacuoles appeared in hepatic cells in the CIH group (Fig. 1a). However, these histological changes partly improved in the CIH + Ad groups. The Oil red O showed the lipid of the hepatic tissue. As shown in Fig. 1b, the lipid level of hepatic tissue in the CIH group were higher than in the NC and NC + Ad groups (all p < 0.05) while the Ad treatment reduced the lipid level compared to the CIH group (p < 0.05). There were no differences between NC and NC + Ad groups in lipid level of hepatic tissue (p > 0.05). The proportions of terminal deoxynucleotidyl transferase dUTP nick-end labeling (TUNEL)–positive cells in the NC (0.896 ± 0.112%) and NC + Ad (0.756 ± 0.109%) groups showed no significant difference (p > 0.05) and were lower than those in other two groups, in which the CIH (9.807 ± 0.474%) group displayed a higher proportion than that in the CIH + Ad (5.167 ± 0.376%) group (all p < 0.05) (Fig. 1c). With ki-67 to detect hepatic proliferation. The proportions of ki-67 positive cells in the NC (0.747 ± 0.094%) and NC + Ad (0.681 ± 0.069%) groups showed no significant difference and both were lower than that in other two groups, in which the CIH (5.066 ± 0.430%) group had a higher proportion than that in the CIH + Ad (3.340 ± 0.304%) group (all p < 0.05) (Fig. 1d).

### ER stress

**Figure 2:**
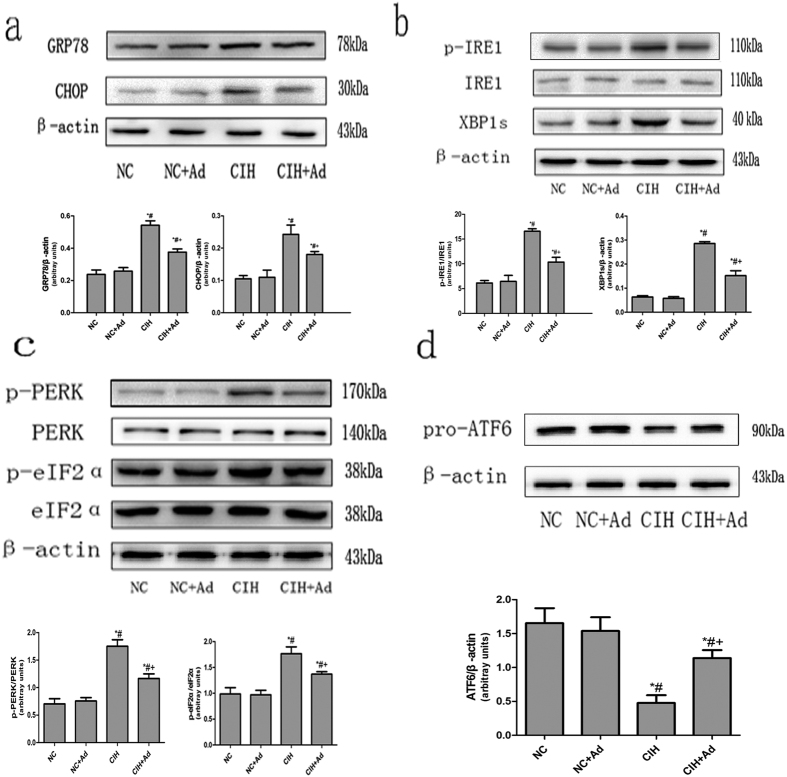
ER stress activation during CIH. (**a**) Protein levels of GRP78 and CHOP in four groups. (**b**) Protein levels of IRE1 pathway. (**c**) Protein levels of PERK pathway. (**d**) Protein levels of ATF6 pathway. The upper panels show the protein levels in four groups. The lower panels show the densitometric evaluation of the independent western blot. N = 3, error bars denote ± S.E.M. *p < 0.05 versus NC; ^#^p < 0.05 versus NC + Ad; ^+^p < 0.05 versus CIH.

To determine whether CIH activated ER stress, we measured the protein levels of GRP78 and CHOP, marker proteins that indicate ER stress. As shown in Fig. 2a, the protein expressions levels of GRP78 and CHOP were higher in the CIH group than in the NC and NC + Ad groups. The protein levels in the CIH + Ad group were remarkably lower than those in the CIH group but higher than those in the NC and NC + Ad groups. These findings were all statistically significant (p < 0.05). There were no significant differences in the protein expression levels between the NC and NC + Ad groups (p > 0.05).

#### IRE1 pathway

To determine whether the IRE1 pathway was activated after CIH, we assessed the hepatic protein expression levels of p-IRE1 and XBP1s by Western blot. As shown in Fig. 2b, the protein levels of p-IRE1 and XBP1s were higher in the CIH group than in the NC and NC + Ad groups. However, Ad supplementation decreased the protein levels, which were still higher than those in the NC and NC + Ad groups. These findings were all statistically significant (p < 0.05). There were no statistically significant differences between the NC and NC + Ad groups (p > 0.05).

#### PERK pathway

To evaluate the activation of the PERK pathway after CIH, we measured the protein levels of p-PERK and p-eIF2α (Fig. 2c). The protein levels of p-PERK and p- eIF2α were higher in the CIH and CIH + Ad groups than in the NC and NC + Ad groups. The protein levels were significantly higher in the CIH group than in the CIH + Ad group. These findings were all statistically significant (p < 0.05). There were no statistically significant differences between the NC and NC + Ad groups (p > 0.05).

#### ATF6 pathway

To evaluate the ATF6 pathway following CIH, we measured the protein level of pro- ATF6 (Fig. 2d). The protein level of pro-ATF6 was lower in the CIH group than in the NC and NC + Ad groups, whereas Ad supplementation increased the protein expression level compared to that of the CIH group. However, the CIH + Ad group also had a lower protein expression level than those of the NC and NC + Ad groups. These findings were all statistically significant (p < 0.05). There were no statistically differences between the NC and NC + Ad groups (p > 0.05).

**Figure 3:**
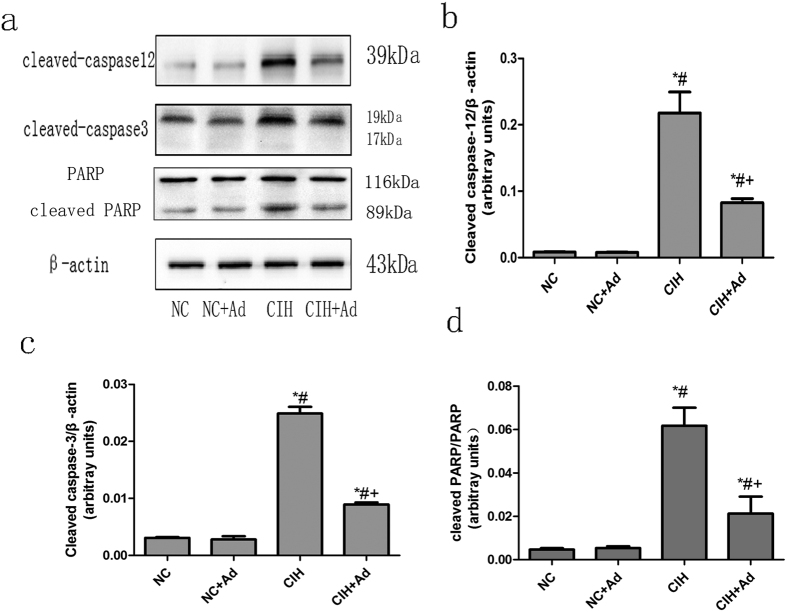
ER stress associated hepatic apoptosis. (**a**) The protein levels of cleaved caspase-12, cleaved caspase-3 and PARP in four groups. (**b–d**) The densitometric evaluation of the independent western blot. N = 5, error bars denote ± S.E.M. *p < 0.05 versus NC; ^#^p < 0.05 versus NC + Ad; ^+^p < 0.05 versus CIH.

The protein levels of cleaved caspase-12, cleaved caspase-3 and PARP (Fig. 3) were higher in the CIH group than in the NC, NC + Ad and CIH + Ad groups, while the protein levels in the CIH + Ad group were also higher than those in the NC and NC + Ad groups. These findings were all statistically significant (p < 0.05). There were no statistically differences between the NC and NC + Ad groups (p > 0.05).

### Mitochondria injury

**Figure 4:**
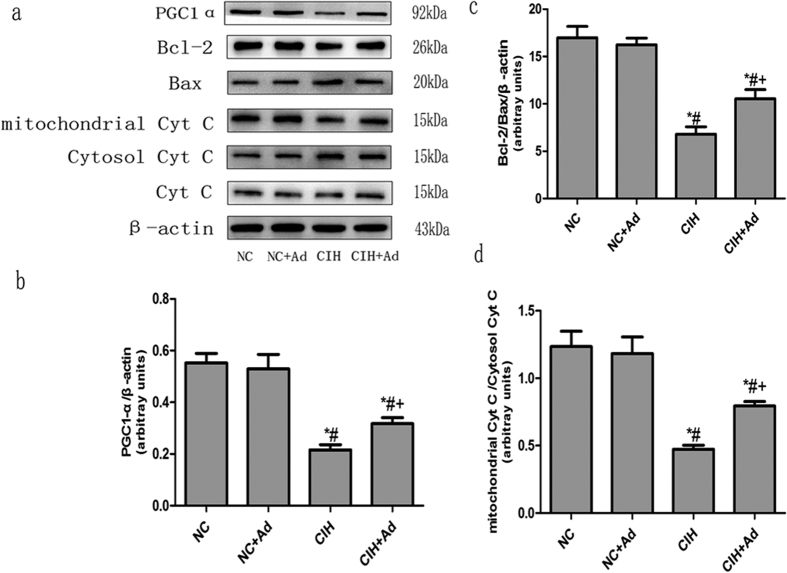
Mitochondria injury. (**a**) The protein levels of BCL-2, Bax, PGC1-α and cytochrome c in four goups. (**b–d**) The densitometric evaluation of the independent western blot. N = 3, error bars denote ± S.E.M. *p < 0.05 versus NC; ^#^p < 0.05 versus NC + Ad; ^+^p < 0.05 versus CIH.

The protein levels of BCL-2, Bax, PGC1-α and cytochrome c were determined to assess the extent of mitochondrial injury (Fig. 4). The protein expression levels of BCL-2/Bax, PGC1α and mitochondria/cytoplasm cytochrome c were all lower in the CIH group than in the NC and NC + Ad groups. The protein levels in the CIH + Ad group were higher than those in the CIH group but lower than those in the NC and NC + Ad groups. These findings were all statistically significant (p < 0.05). There were no statistically significant differences between the NC and NC + Ad groups (p > 0.05).

### Death receptor pathway

**Figure 5:**
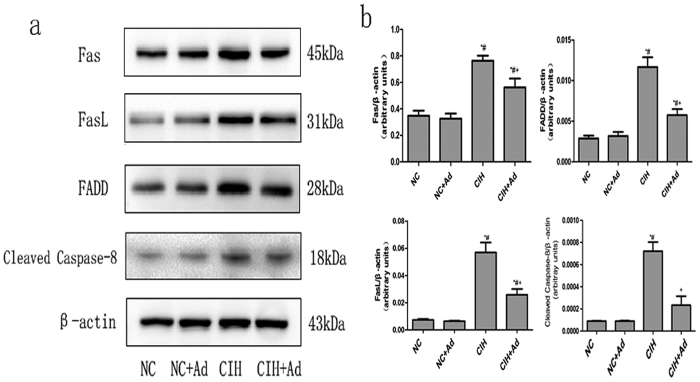
Death receptor pathway. (**a**) The protein levels of Fas, FasL, FADD and cleaved caspase-8 in four groups. (**b**) The densitometric evaluation of the independent western blot. N = 3, error bars denote ± S.E.M. *p < 0.05 versus NC; ^#^p < 0.05 versus NC + Ad; ^+^p < 0.05 versus CIH.

To assess the activation of death receptor pathway, the protein levels of Fas, FasL, FADD and cleaved caspase-8 were determined. As shown in Fig. 5, all the protein levels were raised in the CIH group compared with the NC, NC + Ad and CIH + Ad groups, whereas the protein levels in the CIH + Ad group were still higher than in the NC and NC + Ad groups. These findings were all statistically significant (p < 0.05). There were no statistically significant differences between the NC and NC + Ad groups (p > 0.05).

### Reactive oxygen species (ROS)

**Figure 6:**
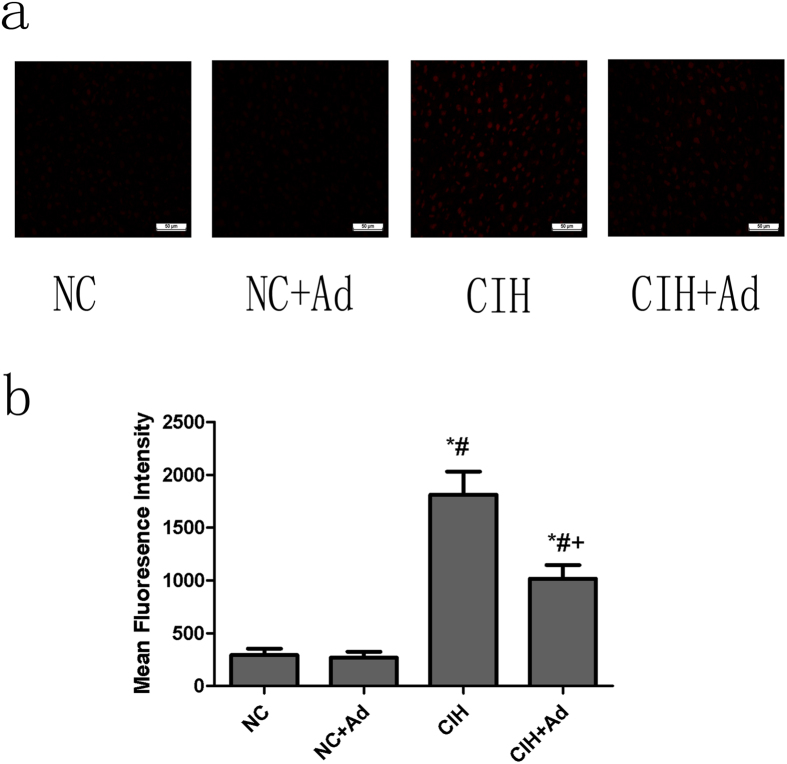
ROS levels. (**a**) The levels of ROS in four groups. (**b**) The densitometric evaluation of the ROS. N = 5, error bars denote ± S.E.M. *p < 0.05 versus NC; ^#^p < 0.05 versus NC + Ad; ^+^p < 0.05 versus CIH.

Dihydroethidium (DHE) staining was used to measure the production of ROS in hepatic tissue (Fig. 6). The intensity of DHE fluorescence was higher in the CIH group than in the NC and NC + Ad groups; however, after supplementation with Ad, the intensity was weaker than that in the CIH group, although it was more intense than that in the NC and NC + Ad groups. These findings were all statistically significant (p < 0.05). There were no differences in DHE fluorescence intensity between the NC and NC + Ad groups (p > 0.05).

### The AMP-activated protein kinase (AMPK) pathway

**Figure 7:**
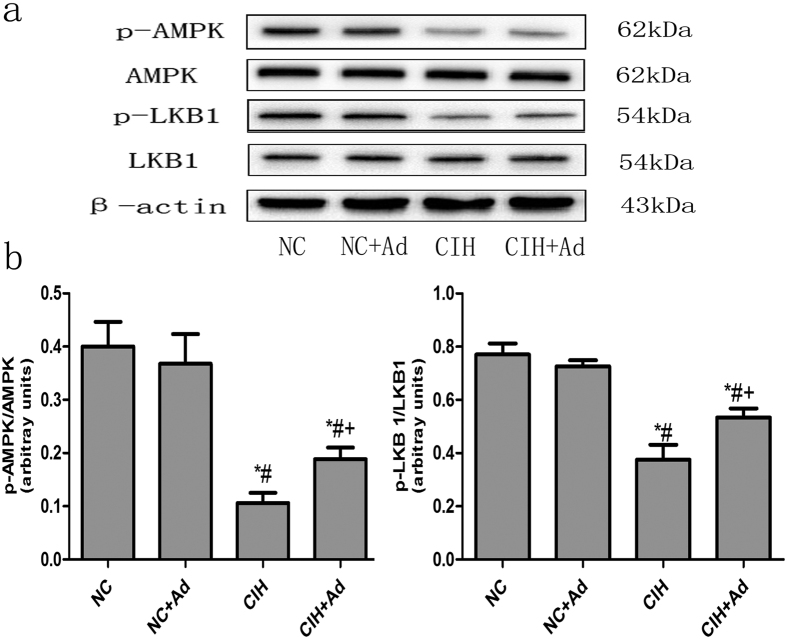
The AMPK pathway. (**a**) The protein levels of p- LKB1 and p-AMPK in four groups. (**b**) The densitometric evaluation of the independent western blot. N = 3, error bars denote ± S.E.M. *p < 0.05 versus NC; ^#^p < 0.05 versus NC + Ad; ^+^p < 0.05 versus CIH.

The AMPK signaling was detected by assessing the protein levels of p-LKB1 and p-AMPK (Fig. 7). After CIH, the protein levels of p-LKB1 and p-AMPK were reduced compared to those in the NC and NC + Ad groups. The protein levels were higher in the CIH + Ad group than in the CIH group (p < 0.05). However, the protein levels in the CIH + Ad group were also lower than those in the NC and NC + Ad groups. These findings were all statistically significant (p < 0.05). There were no statistically statistic differences between the NC and NC + Ad groups (p > 0.05).

## Discussion

To assess the effect of Ad on the liver after CIH, we established a rat protocol that mimicked the CIH conditions present in OSAS. In this study, we found that CIH could increase the liver lipid and triglyceride, FBG and the serum levels of AST, ALT and LDH, ALP and triglyceride, reduce the FSI, and induce hepatic cell apoptosis. The hepatic cell proliferation failed to compensate for the hepatic cell apoptosis. ROS-related ER stress, mitochondrial injury and activation of death receptor pathway might be responsible for hepatic injury, and Ad treatment could mitigate hepatic injury through activation of the AMPK pathway. Further study is needed to prove and elucidate our hypothesis by the block or activating procedures.

CIH has been reported to be a crucial factor in the development of NAFLD and people with OSAS, which is a CIH condition, have an increased risk of NAFLD development^8^,^27^. Sing *et al.* reported that of 190 patients with NAFLD, 46% of the patients with high aminotransferase levels had OSA symptoms^28^. Some reports have shown that the presence of severe OSA was associated with a greater degree of steatosis and necrosis on hepatic biopsy. In addition, CIH aggravated intrahepatic endothelial dysfunction in cirrhotic rats^10^,^29^. Chin *et al.* showed that 35% of 44 OSA patients had elevated AST and ALT levels^30^. Savransky, V *et al.* found that 12 weeks CIH exposure could elevate serum ALT, triglyceride, FBG, reduce FSI in Lean C57BL/6J mice without obesity, but keep the AST, LDH, ALP and hepatic triglyceride unchanged^11^. In our study, we found that the serum levels of hepatic enzymes (ALT, AST, LDH and ALP), serum triglyceride, hepatic triglyceride, FBG were elevated and the FSI was decrease in rats after CIH. The differences species, feed environments and processing may be contributed to the different results between our and the Savransky’s studies. Based on these findings, it was strongly suggested that hepatic injuries could be the result of CIH.

To evaluate the mechanism of hepatic injury induced by CIH, we measured the extent of hepatic cell apoptosis using TUNEL staining. The results showed that the proportion of TUNEL – positive hepatic cells were both increased after CIH. We therefore speculated that hepatic apoptosis might be responsible for the abnormal hepatic enzyme levels. Currently, there are three classic pathways that lead to apoptosis: the mitochondrial pathway, the ER pathway and the death receptor pathway^13^,^31^,^32^,^33^,^34^. In this study, we focused on all three pathways.

This study showed that GRP78 and CHOP, two markers of ER stress, were activated by CIH. In addition, the IRE1, PERK, and ATF6 pathways were all activated after CIH. ER stress is a protective response to cellular stimulation; however, when the stimulation is excessive, the UPR will lead to cell apoptosis^15^,^35^. Once the UPR is activated, the p-IRE1 protein activates its downstream effector, XBP-1, which in turn activates CHOP. A second pathway is mediated by PERK, which activates p-eIF2α, which then activates CHOP. The third pathway is mediated by ATF6, which can also activate CHOP^36^. Evidence from multiple studies showed that CHOP is associated with cell apoptosis^37^,^38^,^39^. In addition to the three apoptotic pathways, ER stress can activate caspase-12, a marker of ER stress associated apoptosis^40^. Caspase-12 can in turn active caspase-3 and caspase-3 further activates the PARP, which then led to cell apoptosis^41^. Consistent with these reports, our experiment found that the protein levels of CHOP, cleaved caspase-12, cleaved caspase-3 and PARP all increased after CIH. Based on these findings, we found that the hepatic apoptosis induced by CIH was associated with ER stress.

CHOP has been reported to regulate the apoptosis-associated proteins localized on the mitochondrial membrane that comprise the BCL-2 family. CHOP reduces the expression of BCL-2 and induces the expression of BIM and Bax^13^,^42^,^43^. These proteins play important roles in mitochondria-mediated apoptotic pathways^44^. Considering the potential relationship between mitochondrial injury and ER stress, we speculated the CIH might also damage mitochondria. To assess the extent of mitochondrial injury, the protein levels of BCL-2, Bax, PGC1-α, and cytochrome c were measured. Consistent with our hypothesis, the protein expression levels of BCL-2/Bax and PGC1-α were decreased after CIH. The cytoplasmic protein level of cytochrome c was increased, but the mitochondrial cytochrome c levels were decreased. These results suggested that mitochondria injury occurs in response to CIH. It is well known that the BCL-2 family is associated with cellular apoptosis^45^,^46^. Therefore, mitochondrial injury may be partly responsible for the hepatic cell apoptosis.

The Fas-FasL signaling is the most popular of the death receptor pathways. Fas-FasL signaling activation can lead to cell apoptosis^47^. Fas-FasL signaling can induce cell apoptosis by activating caspase-8, which further activates caspase-3^48^. Our study found after CIH exposure, the Fas-FasL signaling was activated. Therefore, the death receptor pathway may be partly responsible for the hepatic cell apoptosis.

Some studies have revealed that ROS can trigger ER stress, activate death receptor pathway and induce mitochondrial injury^49^,^50^,^51^. Wang *et al.* have reported that in the OSAS rodent model, CIH caused the production of ROS *in vivo*^52^. Therefore, we speculated that CIH activated three apoptosis pathways through induction of ROS production in hepatic tissue. In agreement with our proposal, our experiment demonstrated that CIH increased the production of ROS in the liver. From this result, we concluded that the production of ROS might be a bridge between CIH and hepatic cell apoptosis.

Our results showed that Ad supplementation, even under CIH conditions, partially ameliorated hepatic injury; Ma, H *et al.* showed that Ad might play an important role in the development of NALFD, and up regulating the level of Ad alleviated the hepatic injury^53^. All of these findings suggested that the Ad played a protective role in hepatic injury. However, the mechanism by which Ad protected the liver against CIH is unknown. Some reports suggested that Ad could inhibit the production of ROS in neutrophils or in the context of myocardial ischemic reperfusion injury^54^,^55^. We found that Ad supplementation decreased the production of ROS, the activation of three apoptosis pathways and the hepatic cell apoptosis. Based on these studies, we speculated that Ad protected the liver partly through inhibition of ROS production, which then inhibits ROS related ER stress, mitochondrial injury and activation of death receptor pathway. AMPK is an important protein kinase which plays a crucial role in the energy balance of mammalian cells and regulates the celluar growth^56^,^57^. Some studies have shown that activation of the AMPK pathway could reduce the ROS levels in cells^58^,^59^,^60^. Studies have also demonstrated that Ad could activate the AMPK pathway, and the AMPK pathway was inhibited in heart tissue in Ad-deficiency mice^61^,^62^. In accordance with some other studies, our study also revealed that the production of ROS reduced while the activation of AMPK pathway increased after the injection of Ad. Our findings suggested that Ad protected the liver partly through inhibition of ROS levels via activation of the AMPK pathway.

In addition, the activation of AMPK pathway could regulate the fat metabolism, increase the glucose uptake in muscle and might be a target for treating diabetes^63^. Yonchu Jenkins *et al.* found that in *db/db* mice the glucose oxidation was enhanced in liver tissue and skeletal muscles through treating with the R419 which can activate the AMPK pathway^64^. S-L Huang *et al.* found the activation of AMPK pathway by Arctigenin could reduce the FBG and serum cholesterol and alleviate glucose tolerance and dyslipidaemia in *ob/ob* mice^65^. These researches showed the activation of AMPK played important roles in glycometabolism and adipose metabolism *in vivo*. In our study, with the supplement of Ad the activation of AMPK was induced, the FBG, hepatic and serum triglyceride as well as the lipid level of hepatic tissue all reduced. Considering all of these, we speculated that Ad might improve the hepatic glycometabolism and adipose metabolism through the activation of AMPK.

In conclusion, our study showed that a) CIH could damage the liver, as evidenced by elevated hepatic enzyme levels, serum triglyceride, liver triglyceride, FBG and enhanced hepatic cellular apoptosis associated with ROS related three apoptosis pathways and b) Ad could protect the liver against CIH, possibly partly by activating the AMPK pathway, then inhibiting the hepatic cell apoptosis. However, further investigations are still needed to ascertain the exact mechanisms by which Ad can protect protection to hepatic tissue against hypoxic insults.

## Materials and Methods

The study was approved by the Animal Ethics Committee of Nanjing Medical University. All experimental protocol including any relevant details were approved by the Animal Ethics Committee of Nanjing Medical University.

The methods were carried out in accordance with the approved guidelines.

### Antibodies and reagents

GRP78, CHOP, PERK, p-PERK, eIF2α, BCL-2, Bax, cytochrome c, caspase-3, p-LKB1, LKB1, p-AMPK, AMPK, PARP and p- eIF2α antibodies were purchased from Cell Signaling Technology (Danvers, MA, USA) and IRE1, p-IRE1, XBP1s,Fas, FasL, FADD, caspase-8 and caspase-12 antibodies were purchased from Abcam (Cambridge, UK) while β-actin, PGC1-α and ATF6 antibodies were purchased from Santa Cruz (CA, USA). Unless otherwise noted, all chemical reagents were purchased from Sigma (St. Louis, MO, U.S.A).

### Animals

A total of 60 male Wistar rats (weight 200–230 g) were purchased from Shanghai Silake Ltd Inc. The rats were housed in the Animal Care Center, which maintained a 12:12 hour light-dark cycle, and were allowed free access to standard chow and water.

### Protocol

We followed a previously reported protocol for CIH^18^. The rats were randomly distributed into four groups of 15 animals: NC, NC + Ad, CIH and CIH + Ad groups. These rats were housed in special cages with a controlled gas delivery system that regulated the flow of air, nitrogen, and oxygen into the cages. The fraction of inspired oxygen (Fi,O2) that was provided to the cages for the CIH group and the CIH + Ad group was decreased from ~21% to ~5–6% and was sustained for 15~20 s over a 1 min period, and then increased to 21% with rapid oxygenation to room air levels in the subsequent 1 min period. The NC and NC + Ad groups received the same gas-flow exposure as that of the CIH and CIH + Ad groups, except only room air was used. The rats in the CIH and CIH + Ad groups were subjected to these intermittent hypoxia events for 8 hours per day for 4 months. In addition, the rats in the NC + Ad and CIH + Ad groups were also treated with an intravenous (IV) injection of Ad at a dosage of 10 μg per injection, twice a week, for 4 successive months. The IV Ad is rat globular adiponectin purchased from Biovision (USA), diluted in PBS, free from endotoxins. Simultaneously, saline (0.5 ml per injection) was injected in the NC and CIH groups. The data were collected at the end of the exposure.

### Tissue and blood samples processing

After the exposure of CIH, the rats were kept fasting for one night. The FBG was detected through tail vein. Then the rats were killed with pentobarbital. The chest was opened for collecting blood and the abdominal cavity was opened for collecting the liver tissues. The liver tissues were stored into −70 °C and 4% paraformaldehyde. After 48 h, the tissues in paraformaldehyde were dehydration and paraffinembedded. Then the paraffinemdded tissues were cut into 5 μm sections. The blood was centrifuged at 3,000 g for 15 min at 4 °C. Then the supernatant (serum) was collected.

### Biochemical determinations

The serum levels of ALT, AST, ALP, LDH, triglyceride and FSI in the rats were measured using an ELISA kit according to the instructions (USCN life, USA). The FBG was detected by One Touch Ultra Glucometer (Roche, Germany). 1 g liver tissues were homogenized with 20 ml chloroform/methanol (volume rate: 2/1). Then the hepatic triglyceride was extracted according to Folch method^65^. The hepatic triglyceride content was detected by using ELISA kit from USCN life.

### ROS analysis

The liver tissues were embedded in optimal cutting temperature compound in ethanol and dry ice and sectioned into 8-μm-thick slices. Then, the sections were immersed in a DHE source (10 μmol/L) in a dark humidified chamber for 30 min at 37 °C. The sections were examined with fluorescence microscopy.

### Histological assay

The frozen sections were stained with HE and Oil red O kit (Jiancheng, Nanjing, China) according to instructions. And the paraffin sections were stained with anti-Ki67 antidody and TUNEL according to the instruction. The anti-Ki67 antibody bought from Abcam was used to detect the hepatic proliferation while the TUNEL kit (Roche, Germany) was used to determine the hepatic cell apoptosis. Histological assessment of liver tissue was performed by using the light microscope (Olympus, Tokyo, Japan).

### Western blot analysis

The total proteins were extracted from the liver tissue with the Tissue Protein Extraction Reagent (Thermo Scientific, USA) containing 1 mM of PMSF (Roche, Germany) and a phosphatase inhibitor cocktail (Roche, Germany), as we have previously described^18^.

The liver cytosolic and mitochondrial fractions were isolated by using the kit (Thermo Scientific, Rockford, USA) as kit instruction. After washing the liver tissue with PBS, the tissues were cut into small piece and infused into mitochondrial isolation reagent A containing 1 mM of PMSF. Use dounce to homogenize the liver tissue and add mitochondria isolation reagent C containing 1 mM of PMSF. The tissues were centrifuged at 700 g for 10 min and supernatant was collected. Then the supernatant was centrifuged at 3,000 g for 15 min. The supernatant (cytosolic fractions) and the mitochondrial pellet were separate collected. Then the mitochondrial was lysed in 2% chaps in Tris-buffered saline (TBS; 25 mM Tris, 0.15 M NaCl; pH 7.2). The cytosolic fractions was used to quantify cytosolic cytochrome c and the mitochondrial was used to detect the mitochondrial cytochrome c.

The protein content was detected by the bicinchoninic acid (BCA) method using a protein assay kit (Thermo Scientific, Rockford, USA). Equal protein amounts were subjected to 10% sodium dodecyl sulfate PAGE and transferred to polyvinylidene fluoride membranes (Roche, Germany). Then, the membranes were incubated with the primary antibodies at 4 °C overnight with gentle shaking, followed by incubation with the peroxidase-labeled secondary antibody for 1 hour at 37 °C. The bands were detected with an enhanced ECL kit (Thermo Scientific, Rockford, USA).

### Statistical analysis

The values are presented as the means ± SEM of three independent experiments. Significant differences between all groups were assessed by one-way analysis of variance using the Student- Newman-Keuls test. A value of p < 0.05 was considered significant.

## Additional Information

**How to cite this article**: Ding, W. *et al.* Adiponectin protects the rats liver against chronic intermittent hypoxia induced injury through AMP-activated protein kinase pathway. *Sci. Rep.* **6**, 34151; doi: 10.1038/srep34151 (2016).

## Figures and Tables

**Table 1:** The serum levels of biochemical changes.

	NC	NC + Ad	CIH	CIH + Ad
Serum AST (U/L)	173.9 ± 13.00	171.5 ± 15.10	333.5 ± 21.84*#	256.3 ± 24.68*#+
Serum ALT (U/L)	49.63 ± 10.79	51.33 ± 11.42	129.2 ± 17.91*#	88.39 ± 10.73*#+
Serum LDH(U/L)	771.9 ± 32.48	833.0 ± 29.19	2435 ± 69.67*#	1577 ± 70.11*#+
Serum ALP(U/L)	111.4 ± 18.24	116.9 ± 16.12	218.1 ± 36.91*#	152.2 ± 18.93*#+
FBG (mmol/L)	4.237 ± 0.368	4.281 ± 0.347	6.695 ± 0.405*#	5.255 ± 0.236+
FSI (mmol/L)	12.40 ± 0.58	12.34 ± 0.66	7.52 ± 0.73*#	9.51 ± 0.35*#+
Serum triglyceride (mmol/L)	0.472 ± 0.033	0.440 ± 0.022	0.699 ± 0.043*#	0.573 ± 0.032*#+
hepatic triglyceride (mmol/L)	0.076 ± 0.002	0.086 ± 0.007	0.150 ± 0.008*#	0.106 ± 0.006*#+

N = 15, ^*^p < 0.05 versus NC; ^#^p < 0.05 versus NC + Ad; ^+^p < 0.05 versus CIH.
